# Artificial intelligence in liver diseases: Improving diagnostics, prognostics and response prediction

**DOI:** 10.1016/j.jhepr.2022.100443

**Published:** 2022-02-02

**Authors:** David Nam, Julius Chapiro, Valerie Paradis, Tobias Paul Seraphin, Jakob Nikolas Kather

**Affiliations:** 1 Section of Interventional Radiology, Department of Radiology and Biomedical Imaging, Yale School of Medicine, New Haven, CT, USA; 2 INSERM U1149 "Centre de Recherche Sur L'inflammation", CRI, Université de Paris, Paris, France; 3 University Paris, AP-HP, Department of Pathology, Hôpital Beaujon, Clichy, France; 4 Department of Gastroenterology, Hepatology and Infectious Diseases, Medical Faculty of Heinrich Heine University Düsseldorf, University Hospital Düsseldorf, Düsseldorf, Germany; 5 Department of Medicine III, University Hospital RWTH Aachen, Aachen, Germany; 6 Pathology & Data Analytics, Leeds Institute of Medical Research at St James's, University of Leeds, Leeds, UK; 7 Medical Oncology, National Center for Tumor Diseases (NCT), University Hospital Heidelberg, Heidelberg, Germany

**Keywords:** Artificial intelligence, deep learning, machine learning, diagnostic support system, imaging, multimodal data integration, AI, artificial intelligence, CNN, convolutional neural network, DICOM, Digital Imaging and Communications in Medicine, HCC, hepatocellular carcinoma, ML, machine learning, MVI, microvascular invasion, NAFLD, non-alcoholic fatty liver disease, NASH, non-alcoholic steatohepatitis, TACE, transarterial chemoembolisation, TRIPOD, Transparent Reporting of a multivariable prediction model for Individual Prognosis or Diagnosis, WSIs, whole slide images

## Abstract

Clinical routine in hepatology involves the diagnosis and treatment of a wide spectrum of metabolic, infectious, autoimmune and neoplastic diseases. Clinicians integrate qualitative and quantitative information from multiple data sources to make a diagnosis, prognosticate the disease course, and recommend a treatment. In the last 5 years, advances in artificial intelligence (AI), particularly in deep learning, have made it possible to extract clinically relevant information from complex and diverse clinical datasets. In particular, histopathology and radiology image data contain diagnostic, prognostic and predictive information which AI can extract. Ultimately, such AI systems could be implemented in clinical routine as decision support tools. However, in the context of hepatology, this requires further large-scale clinical validation and regulatory approval. Herein, we summarise the state of the art in AI in hepatology with a particular focus on histopathology and radiology data. We present a roadmap for the further development of novel biomarkers in hepatology and outline critical obstacles which need to be overcome.

**Key points**

• Clinical decision making in hepatology relies on a diverse set of data modalities.
• Classical machine learning tools such as random forests and deep learning tools such as convolutional neural networks can extract clinically useful information from complex data.
• In particular, histopathology and radiology images of liver diseases contain a wealth of information.
• A number of proof-of-concept studies have demonstrated the usefulness of these methods in hepatology.
• Future efforts from academic and industry partners are required to establish machine learning and deep learning tools in the clinical practice of hepatology.

## Introduction

### Hepatology - a complex art

Hepatology is the clinical study of liver disease and is a prime example of the complexity of modern medicine. To diagnose disease, make a prognosis about disease outcomes, and recommend an optimal treatment, clinicians rely on a vast array of diagnostic data modalities. The standard clinical workup of patients with suspected or confirmed liver disease includes taking the clinical history, performing a clinical examination, running laboratory tests, and interpreting imaging studies. Liver biopsies may even be performed, requiring assessment of changes in tissues, cells and molecular markers. Collectively, these data modalities contain a wealth of information. Interpretation of this information is a challenging task, even for seasoned clinicians, and diagnostic ambiguities abound in hepatology.^1^

### Machine learning and deep learning

Artificial intelligence (AI) enables computers to learn from complex datasets and solve real-world problems within and beyond medicine, leading to performances on par with or better than those of their human counterparts. AI refers to computational approaches to data analysis in which computer programmes are not explicitly guided by experts but primarily learn from examples. Throughout this article, we will use AI as a broad term that includes classical machine learning (ML) and deep learning (DL) techniques.^2^ **Classical ML techniques** do not require dedicated hardware and have been used for decades in medicine, including hepatology and gastroenterology studies.^3^ These techniques rely on “handcrafted features” defined by human investigators. What does this mean in the context of hepatology? An example of AI as applied to hepatology is automatic prognostication of solid tumours based on imaging data. Using a handcrafted approach, human investigators assemble a list of quantitative visual features such as tumour size, roundness, symmetry and intensity on images.^4^ These features are subsequently inputted into a classification algorithm, for example, the “random forest” method, which excels at categorising such tabular data.^5^ In radiology image analysis, handcrafted image analysis approaches are traditionally termed “radiomics” (or “classical radiomics”). In addition to this established ML approach, **“deep learning” (DL)** has blossomed in the last 10 years thanks to algorithmic advances, improved hardware, and large datasets. While conceptually similar to classical ML approaches, DL methods usually have thousands more free parameters than classical ML methods. This abundance of parameters makes DL models more flexible and better suited for processing and classifying complex data sets such as language data or imaging data. In medicine, the most commonly used DL methods are artificial neural networks (used for image processing^6^ and processing of time series^7^) and transformers (used for language processing^8^ and, more recently, image processing^9^). Importantly, in a DL approach, investigators do not assemble lists of handcrafted features. Rather, a DL network is entrusted with automatically finding features associated with an endpoint, specifically the clinical outcome. Given today’s technologies, DL methods usually outperform handcrafted feature-based approaches and consequently dominate the field of AI in hepatology. However, the demarcation between handcrafted approaches and DL is not absolute; multiple studies have used DL systems to extract features, which are subsequently combined with handcrafted features.^10^^,^^11^ Application-wise, ML/DL approaches can be used for two ends. First, they can recapitulate, and thus automate, the interpretation of data normally performed by human experts. Second, they can extract subtle features from complex data which are not immediately obvious to the human eye.^12^

### Academic research on AI in hepatology

**Fig. 1:**
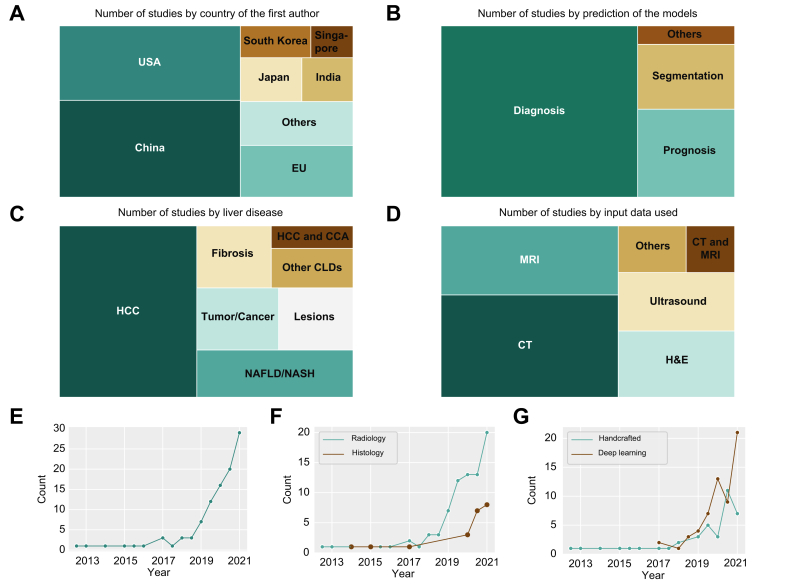
Digital pathology and radiology using artificial intelligence for management of liver diseases. (A) Number of studies by country of the first author. (B) Number of studies by prediction of the models. (C) Number of studies by liver disease. (D) Number of studies stratified by the clinical input data used. Raw data for this figure is available in Tables S1 and S2. Methodological details are available in the supplementary materials and methods. (E) Cumulative number of published original studies per half-year from 2010 to mid-2021. (F) Cumulative number of published original studies per half-year by research field. (G) Cumulative number of published original studies per half-year by either deep learning or handcrafted feature extraction. CCA, cholangiocarcinoma; CLD, chronic liver disease; HCC, hepatocellular carcinoma; NAFLD, non-alcoholic fatty liver disease; NASH, non-alcoholic steatohepatitis.

Academic research groups from multiple countries are actively engaged in ML/DL research in hepatology. Based on a quantitative survey of the MEDLINE database (supplementary information), researchers from China and the USA are the most prolific, with between 30 and 40 total publications on ML/DL in hepatology (Fig. 1A). By far the most common application is automatic diagnosis of liver disease from imaging data (Fig. 1B). In these cases, the ground truth is derived from the image data itself. For instance, an expert radiologist diagnoses a malignant liver mass in a CT dataset and the ML/DL algorithm is tasked with reproducing this diagnosis in a supervised training experiment. Another group of studies involves prognosis prediction from image-based data. Forecasting the natural course of a disease can have direct implications for the clinical management of patients. Accurate prognostication allows clinicians to adjust follow-up intervals, convey the urgency of lifestyle changes to patients, and adjust the intensity or type of pharmacological treatment. A third category of applications is segmentation of structures of interest. Segmentation studies aim to generate an accurate outline around a region of interest. As a clinical example, algorithms can delineate organs at risk before radiation therapy of cancer. While ML/DL studies in hepatology address a range of diseases, almost all published studies address either neoplastic or metabolic diseases of the liver, which are the major causes of liver-related morbidity and mortality besides viral hepatitis^13^ (Fig. 1C). ML/DL studies in hepatology currently incorporate a range of imaging modalities. The 3 most commonly analysed modalities are CT scans, MRI scans and H&E-stained histopathology slides (Fig. 1D). In the last 4 years, the number of ML/DL studies in hepatology has exponentially grown (Fig. 1E), even more so in radiology than in histopathology (Fig. 1F), and only 1 study has combined both data modalities so far.^14^ In addition, a trend toward a larger growth of DL studies compared to handcrafted feature-based studies can be observed (Fig. 1G).

### Implementation of AI in hepatology

At this point, a number of ML/DL tools are already approved for clinical use by the US FDA and similar regulatory agencies worldwide.^15^ Nevertheless, there is a wide gap between the burgeoning number of research articles and the limited number of clinically approved, available applications. This discrepancy is exacerbated by missing external and prospective validation of models, lack of technological infrastructure in health facilities, lack of knowledge and trust in ML/DL systems amongst medical personnel, as well as data privacy issues.^16^^,^^17^ Furthermore, the clinical implementation of ML and DL methods in hepatology lags far behind that in other fields of medicine. Recently, the first ML/DL algorithms for management of patients with liver diseases were clinically approved in Europe and the US. In contrast, ML/DL algorithms have already been available in other areas of medicine for a few years, such as polyp detection in colonoscopy, fracture detection in X-ray images and brain volume quantification in magnetic resonance scans.^15^ This is possibly due to the complex nature of hepatology, which rarely depends on a single data type for diagnosis and clinical management. In the following sections, we will review the current progress of ML/DL in hepatology from clinical and technical perspectives, focusing on histopathology and radiology image analysis.

## AI in liver histopathology

### State of the art

#### Challenges in liver histopathology

One of the key challenges in liver histopathology is the clinical decision to obtain liver tissue via biopsy. While liver biopsy is a safe procedure for most patients, it is associated with non-negligible morbidity. Moreover, national guidelines and clinical practice are not always consistent about when a biopsy’s benefits outweigh its risks.^18^ This explains the obvious need for non-invasive biomarkers and likely explains the abundance of ML/DL studies in liver radiology (Fig. 1F). Nevertheless, once a biopsy has been obtained, there is a clinical need for a fast, definitive, reliable, reproducible and quantitative diagnosis.^19^ It was not until 2020 that the application of ML/DL methods in liver histopathology gathered pace. Unlike radiology which adopted radiomics in several studies, histopathology did not extensively apply ML methods using handcrafted features. Rather, most research groups immediately adopted emerging DL algorithms based on convolutional neural networks (CNNs), which were originally developed for non-medical computer vision tasks.

#### Diagnosis and segmentation in fatty liver disease

Most studies in histopathology have used data (whole slide images [WSIs]) from patients with non-alcoholic fatty liver disease (NAFLD), non-alcoholic steatohepatitis (NASH) or hepatocellular carcinoma (HCC) (Table S1). All of these diseases share the clinical need for clear-cut diagnostic and prognostic systems. Several studies have focused on models quantifying steatosis, inflammation, hepatocellular ballooning and other morphological patterns in patients with NAFLD, as well as the staging of liver fibrosis.[20], [21], [22] In 2014, Vanderbeck *et al.* published one of the first studies using handcrafted features in a support vector machine algorithm to identify and quantify macrosteatosis, central veins, bile ducts and other structures on scanned H&E slides from NAFLD and healthy liver biopsies, with an overall accuracy of 89%.^23^ In the following year, the same group extended their algorithm for the classification of lobular inflammation and hepatocyte ballooning with AUCs of 0.95 and 0.98, respectively. Another study developed a ML quantifier of morphological features of NAFLD to calculate a diagnostic score for NASH, yielding an AUC of 0.80 (95% CI 0.68-0.89).^24^ Applying classical ML techniques, Leow *et al.* used unstained liver biopsies and second-harmonic imaging microscopy to stratify stage 1 and 2 NASH fibrosis.^25^ Roy *et al.* developed an algorithm with a U-Net architecture which adequately segmented and quantified hepatic steatosis.^26^ Another benchmark study in the field of quantifying morphological features and staging of fibrosis in NASH biopsies was conducted by Taylor-Weiner *et al.*, who developed and validated their models retrospectively on 3 patient cohorts from large randomised controlled trials. Their quantifications correlated with the assessment of 3 experienced pathologists. Specifically, the feature outputs of their model were able to predict disease progression in patients with NASH, with C-indices of up to 0.73.^27^ Gawrieh *et al.* designed a model to quantify fibrosis in trichrome-stained biopsies of patients with NASH, achieving good correlation with pathologists’ assessments. Additionally, their model was able to classify different patterns of fibrosis with AUCs between 0.77 and 0.95.^28^ Overall, these studies show the potential of ML/DL technology for segmentation, quantification and standardisation of diagnosis in patients with NAFLD and NASH.

#### Diagnosis and segmentation in primary liver cancer

In recent years, multiple studies have generated AI models for classifying, segmenting and diagnosing tissue from HCC samples.[29], [30], [31] Li *et al.* published a CNN-based DL algorithm that was able to grade HCC nuclei on liver histopathology, while Lal *et al.* published a more complex model to fulfil the same task 4 years later in 2021.^32^^,^^33^ Wang *et al.* developed a DL model which accurately identified tumour tissue in hyperspectral data of unstained HCC samples.^34^ Sun *et al.* used the DL technique of multiple instance learning to distinguish between HCC and normal liver tissue in WSIs, reporting AUCs of nearly 1.00.^35^ Using a convolutional autoencoder, Roy *et al.* detected tumour tissue and segmented WSIs.^36^ Some of the challenges of adopting a medical AI-assistance tool were highlighted by Kiani *et al.*, who trained a CNN on image patches from H&E slides of hepatic tumours to distinguish between HCC and cholangiocellular carcinoma with a slide level accuracy of 0.88 (95% CI 0.71–0.96). Subsequently, the model’s performance as an assistive tool for 11 pathologists with different experience levels was evaluated. The results showed that even though it did not significantly improve the accuracy of diagnosis for the whole group of pathologists, the tool improved the accuracy for a subgroup. It also showed that a false prediction of the tool had a negative influence on the pathologist’s decision.^37^ Further development and validation of the findings of these proof-of-concept studies will be needed before their implementation into clinical workflows.

#### Outcome prediction for liver disease

While the previously described studies focused on models imitating human tasks in histopathology, some recent studies have tried to infer clinical endpoints directly from histopathology images. As such, Liao *et al.* developed an image segmentation pipeline capable of distinguishing HCC from healthy liver tissue with an AUC of 0.87 on an external dataset and calculated a risk score associated with overall survival after resection in patients with HCC, facilitating a significant separation of high- and low-risk patients’ Kaplan Meier survival.^38^ A group from Japan used handcrafted features from nuclei segmentation to predict early recurrence after resection of HCC with an accuracy of nearly 0.90.^39^ The capability of DL algorithms to predict survival of patients with HCC from H&E-stained WSIs was impressively shown by Saillard *et al.*, in which a DL risk score outperformed common clinical, biological and pathological features; the American Joint Committee on Cancer staging system; and a composite score of all these variables.^40^ Histopathology’s potential for predicting survival was further corroborated by Shi *et al.*’s DL model, where a “tumor risk factor” was an independent predictor of overall and recurrence-free survival in multivariable analysis adjusted for known prognostic factors in patients with HCC.^41^ Yamashita *et al.* created a risk score showing independent association with recurrence-free survival in patients with HCC who underwent cancer resection.^42^ Applying new techniques of multimodal data input, He *et al.* combined histopathology, MRI, and clinical data to train a model that predicted the risk of HCC recurrence in patients after liver transplantation (AUC of 0.87).^14^ These promising studies are just the tip of the iceberg in an emerging field of research that seeks to find better prognostic markers for clinical endpoints and to harness the potential of digitised histopathology images to support physicians in their clinical decision making.

### What is missing

#### Standardisation of image analysis

In histopathology, a wave of digitisation is expected to occur in the next 5 to 10 years.^43^ However, most diagnostic pathology departments still rely on manual handling of glass slides. Once routine workflows are digitised, DL-based biomarkers can be inexpensively added. However, universal standards for data formatting, image data compression, and storage of metadata do not exist for digital histopathology WSIs. Currently, the field is dominated by vendor-specific data formats, which are similar to multichannel TIFF images and store high-resolution image data in a pyramidal way. This is in stark contrast to radiology, where the Digital Imaging and Communications in Medicine (DICOM) format is the standard for storing image data and metadata, providing a firm ground for the discovery of biomarkers.

#### Diversity and bias in database curation

The performance of AI systems in histopathology generally increases with the number of patients,^44^^,^^45^ while the generalisability of such systems increases with the diversity of patients in the training set.^46^ In the field of cancer research, including HCC, The Cancer Genome Atlas (TCGA) database provides publicly available histologic, genetic, and clinical data on thousands of patients and has served as a key resource for early studies on DL-based biomarkers in HCC.^10^^,^^47^ However, recent studies have uncovered potential biases in the TCGA database leading to overperformance of DL systems.^48^ Therefore, external validation of TCGA-derived classification systems is crucial for generalisability.^16^

### The next steps

Optimistically, ML/DL systems could help resolve the diagnostic, prognostic and predictive issues that limit liver histopathology image analysis. This would improve and facilitate clinical trials in liver disease in which inclusion criteria, patient strata and histological endpoints are often manually defined by pathologists and therefore subject to intra- and inter-observer variability.^49^ As in other disease contexts, there is a place in clinical decision making for invasive tissue-based diagnostics. ML/DL approaches could conceivably improve the consistency, quality and amount of information which researchers and healthcare providers can extract from this tissue. The benefits of these ML/DL approaches to histopathological analysis may incentivise patients to undergo an invasive procedure such as liver biopsy. However, for some problems in the management of liver disease, non-invasive radiology images, instead of invasive diagnostics, can be analysed to unveil biomarkers. In the following section, we will review the state of the art in ML/DL approaches applied to such radiology data.

## AI in liver radiology

### State of the art

#### Challenges in liver radiology

Patients with liver disease, particularly those with liver cancer, undergo multiple imaging studies to establish a diagnosis, pre-operatively plan interventions, and monitor response to therapy (Table S2). Each of these imaging studies contain numerous data points that could be potentially analysed to improve predictions. However, there is a formidable challenge in transforming this burden of clinical and imaging data into something of clinical value.

This challenge in image interpretation is confounded by several considerations. There are at least 25 guidelines for HCC diagnosis with varying, inconsistent definitions for imaging features. Although LI-RADS is the most standardised^50^ of these guidelines, there is no unified imaging guideline that encompasses a patient’s journey from diagnosis and treatment recommendations to therapeutic response assessment. Similarly, treatment recommendations for patients can be inconsistent amongst HCC prognostic staging systems depending on functional status, tumour imaging characteristics, liver function, and geography.[51], [52], [53] In addition, several locoregional and systemic therapies exist,^54^^,^^55^ each of which may introduce distinctive appearances on follow-up imaging.^56^ Finally, ultrasound and elastography are used to non-invasively assess steatosis and fibrosis, but the calibration and discriminative accuracy of these modalities vary greatly.^57^

**Fig. 2:**
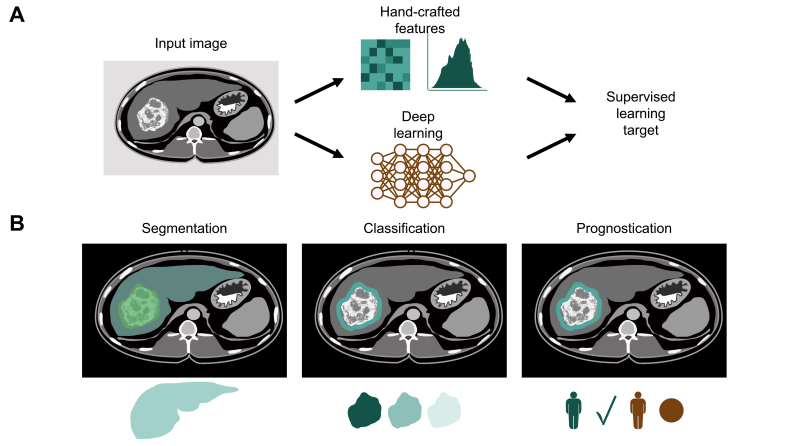
Radiology image analysis workflows. (A) Handcrafted feature extraction, also referred to as “radiomics,” is an established image analysis technique in radiology image analysis. Alternatively, deep learning, in the form of neural networks, can be used to learn features and predict a target label in a supervised fashion (end-to-end analysis). (B) Common tasks in radiology image analysis are segmentation, classification and prognostication.

To facilitate transformation of imaging data into clinically accessible information, AI may derive predictions in a more personalised fashion. Two categories of AI that have shown promise in liver imaging are radiomics (relying on classical ML) and DL systems (relying on CNNs) (Fig. 2A). Radiomics is a strongly supervised and expert-guided approach where hard-coded algorithms extract quantitative image features that are fed into an ML algorithm.^58^ In contrast, DL with a CNN constitutes an automatic feature extraction where the algorithm self-learns salient features and self-optimises parameters by running an input image through mathematical operations embedded in multiple layers.^59^ Because both approaches aim to predict a pre-defined “ground truth,” they are considered supervised learning approaches. Herein, we review AI tools for liver imaging in segmentation, classification of disease severity and lesions, and outcome prediction.

#### Segmentation of liver and liver lesions

Segmentation involves drawing boundaries of the entire organ, a lesion, or other structures of interest on an imaging study (Fig. 2B). CNNs employing a U-Net architecture have been utilised extensively in the medical imaging literature for segmentation tasks.^60^ Namely, Christ *et al.*'s landmark study used a combination of cascaded CNNs with U-Net architectures and dense 3D conditional random fields to determine segmentation of the whole liver and liver lesions on abdominal CT.^61^ While not based on a U-Net architecture, Sun *et al.* used a CNN based on multi-phase contrast-enhanced CT images to segment liver tumours.^62^ To enable head-to-head comparisons of segmentation algorithms, the Liver Tumor Segmentation Benchmark (LiTS) supplied a public dataset of liver CTs and showed that algorithms could achieve segmentation of livers and tumours with Dice scores greater than 95% and 70%, respectively.^63^ A noteworthy example that excelled in lesion segmentation on the LiTS dataset is the H-DenseUNet, a hybrid U-Net fusing 2D intra-slice and 3D inter-slice features.^64^ DL for liver and HCC segmentation can be further refined by excluding false positive segmentations using a radiomics-based random forest and thresholding of mean neural activation.^65^ Practical studies of segmentation include delineation of ablation zones^66^ and anatomy-guided multimodal registration of the liver from MRI to intraprocedural cone-beam CT for locoregional therapy.^67^

#### Tissue characterisation of fibrosis and liver lesions

CNN classification tools may potentially replace liver biopsy for grading the severity of NAFLD and liver fibrosis in some patients (Fig. 2B). CNNs were initially used to classify the presence of fatty liver disease with AUCs of almost 1.00.^68^^,^^69^ Since then, CNNs have been applied for quantification of liver steatosis on abdominal CT screening^70^ and ultrasound.^71^ CNNs classified F3 and F4 fibrosis on 2D shear wave elastography^72^ and portal venous phase CT images^73^ with AUCs of at least 0.95, outperforming the AST-to-platelet ratio index and the fibrosis-4 index. Gadoxetic acid-enhanced hepatobiliary phase MR images have also been inputted into a CNN for fibrosis staging, achieving AUCs of 0.84, 0.84, and 0.85 for classification of F4, F3, and F2 fibrosis, respectively.^74^

CNNs also excel in classification of liver masses. Yasaka *et al.*’s DL CNN model used multi-phase contrast-enhanced CT to diagnose 5 categories of malignant and benign liver masses with a median accuracy of 0.84. The AUC for differentiating HCCs and other malignant lesions *vs.* indeterminate and benign masses was 0.92.^75^ Hamm *et al.* developed a CNN system based on multiphasic MRI that identified 6 classes of hepatic lesions with an AUC of 0.99 for test cases and a sensitivity and specificity (90%/98%) that exceeded that of radiologists (82.5%/96.5%).^76^ For challenging HCC diagnoses, Oestmann *et al.* trained a DL model with multiphasic MRI to differentiate HCC with typical and atypical appearances from non-HCC lesions.^77^

#### Outcome prediction for malignant disease

Given its association with high rates of recurrence after HCC resection, microvascular invasion (MVI) has been the focus of predictive radiomics nomograms. Nomograms using contrast-enhanced CT radiomics signatures yielded AUCs ranging from 0.80 to 0.90 during validation.[78], [79], [80] Notably, Xu *et al.* showed that although radiomic features did not add additional benefit to radiologist scoring of HCC, the integrated nomogram of radiomics, clinical factors, and radiographics achieved an AUC of 0.90 in the test set for predicting MVI.^80^ Feng *et al.* used radiomics features on preoperative Gd-EOB-DTPA (gadolinium ethoxybenzyl-diethylenetriaminepentaacetic acid)-enhanced MRI to predict MVI for curative hepatectomy with an AUC of 0.85 in the validation cohort.^81^ Recent DL models on CT^82^ and contrast-enhanced MRI^83^^,^^84^ can predict MVI with AUCs exceeding 0.90.

Finally, AI has found utility in predicting response to transarterial chemoembolisation (TACE). Abaijian *et al.* used MRI imaging features and clinical variables to develop logistic regression and random forest models that predicted response to TACE.^85^ Morshid *et al.* trained 2 CNNs to segment the liver and HCC, extracted textures from segmented HCCs, and used a random forest to classify patients as being susceptible or refractory to TACE using the extracted textures and the BCLC score.^86^ A residual CNN was utilised in transfer learning to predict RECIST response to TACE based on pretreatment CT images of intermediate stage HCC, with AUCs above 0.90 in independent validation cohorts.^87^ Jin *et al.* created a nomogram of clinical features, radiological characteristics, and a pretreatment CT radiomics signature to predict extrahepatic spread and MVI in patients with HCC who underwent TACE.^88^

### What is missing

#### Standardisation of image analysis

Despite AI’s promise for translation in liver imaging, discrepancies in methodology prevent incorporation into clinical decision making. Considerable variation exists within the radiomics workflow starting from data acquisition to final selection of features,^58^^,^^83^ although similar considerations apply to DL. In liver imaging, CT, MRI, or ultrasound constitute imaging modalities with distinct data acquisition parameters. As such, the use of specific scanners, imaging protocols, and image reconstruction methods could affect later extraction of features.^58^^,^^69^^,^^81^^,^^89^ While most imaging data is stored in a PACS (Picture Archiving and Communication System) as DICOM files, further variability is introduced when files are converted to user-friendly versions such as PNG, TIFF, and NIFTI (Neuroimaging Informatics Technology Initiative).^90^

Difficulties in standardisation also arise during image analysis. Little unity exists around segmentation methods from various vendors,^58^ while DL-based segmentation methods diverge in their architectures. As for preparation of imaging data for feature extraction, image processing steps such as interpolation, normalisation, and discretisation depend on imaging modality, which may affect the reproducibility of radiomic features.^58^ Finally, heterogeneity exists amongst in-house software used for feature selection and dimensionality reduction.

#### Diversity and bias in database curation

In order for AI algorithms to be widely applicable beyond their initial training and validation phases, well-curated databases are crucial for external validation. It is critical to generate an epidemiologically diverse dataset to ensure all imaging appearances are included. For instance, an algorithm developed for fibrosis staging in an East Asian population, where patients predominantly have chronic hepatitis B, may not be generalisable to Western populations, where NAFLD and alcohol-related liver disease are common. In addition, class imbalance in non-diverse datasets can compromise generalisability by negatively affecting the algorithm’s ability to classify test cases that were less represented during the training phase. This could explain why an algorithm may less effectively classify F2 fibrosis, as more advanced F3 and F4 stages are over-represented.^72^^,^^73^

External validation may also be compromised when a dataset unintentionally perpetuates existing disparities in healthcare through the labels it chooses for prediction. This very issue was highlighted in a commercial algorithm that used predicted cost as the algorithmic risk score. At a given algorithmic risk score, Black patients had a higher number of active chronic conditions than White patients, but similar actual, realised costs to White patients. This discrepancy suggested less health spending was allocated to Black patients for their true illness burden, possibly due to barriers in care experienced by Black patients not captured by predicted cost.^91^

### The next steps

Concrete steps can be taken to standardise data collection and image analysis. The Quantitative Imaging Biomarker Alliance (QIBA) has sought to standardise the measurement and analysis of Quantitative Imaging Biomarkers (QIBs) by drafting QIBA profiles dedicated to certain QIBs, whereas the European Imaging Biomarker Alliance has tabulated organ systems-based inventories detailing the evidence for biomarkers.[92], [93], [94] With respect to radiomics, workflows can adhere to the Radiomics Quality Score and Transparent Reporting of a Multivariable Prediction Model for Individual Prognosis or Diagnosis (TRIPOD) statements to ensure technical rigor and verify features are consistent with the Image Biomarker Standardisation Initiative Reference Manual.[95], [96], [97] In addition, publicly sharing details of algorithm development would foster mutual agreement on imaging formats and annotations,^90^ establish benchmarks for methodologies, and facilitate comparisons amongst studies. Finally, AI algorithms should be tested on prospectively collected data to assess the robustness of features in the face of new data.

To supply the diversity of images needed to represent all possible pathologies, multi-institutional databases should be established. Datasets should include multiple geographic regions, provide data from different imaging vendors, and reflect the racial and socioeconomic diversity of the population the AI algorithm will be implemented upon.^90^ Strategies such as data augmentation or general adversarial networks can also be used to expand the dataset and compensate for under-represented classes of images.

Anticipating sources of bias which threaten the external validity of an algorithm will involve pre-emptively acting on biased predictions. AI algorithms should employ continuous, real-time learning in which new input data are monitored for biases^98^ and predicted labels are modified accordingly in external testing to minimise bias.^90^

Sharing all details of algorithm development, especially the datasets and computer source code underlying the model, will be critical for reproducibility, validation, and eventual translation into clinical workflows.[99], [100], [101] The Checklist for Artificial Intelligence in Medical Imaging (CLAIM) and the assessment checklist developed by the Fairness, Universality, Traceability, Usability, Robustness and Explainability AI (FUTURE-AI) initiative establish reporting guidelines for appraisal of AI studies.^102^ Similarly, the checklists for established reporting guidelines, such as STARD (Standards for Reporting Diagnostic Accuracy), CONSORT (Consolidated Standards of Reporting Trials), and TRIPOD, are being expanded specifically to account for ML and AI applications.^103^^,^^104^ The Evaluating Commercial AI Solutions in Radiology (ECLAIR) Guidelines expand upon the aforementioned checklists for AI studies by adding considerations related to information technology infrastructure, user accessibility, medical device regulation, data protection, licensing, and product maintenance.^105^ With greater adherence to reporting guidelines, AI will be able to clearly define its roles in hepatology clinical workflows. Indeed, AI can potentially facilitate triage of patients, enhance consult evaluations, or conveniently summarise all patient clinical data under a single clinical interface.^12^ Moreover, standardisation of AI tools will be needed to encourage the adoption of more clinically relevant performance metrics such as classification/re-classification accuracy and quality of life measures, rather than indices such as the AUC.^16^^,^^105^

Finally, holding algorithms accountable for their predictions may involve proactively ensuring that clinicians understand how algorithms use input data to make decisions, or interpretability. Visualisation methods mapping which pixels contribute to the classification of an input image can aid interpretability of DL systems.^106^ Wang *et al.* worked within the framework of Hamm *et al.*’s DL system to infer features most relevant to hepatic lesion classification and produce feature maps corresponding to areas where features were detected.^76^^,^^107^ Zhen *et al.* generated saliency heatmaps to visualise pixels most relevant to classification of 7 types of focal liver lesions on MRI.^108^ Wei *et al.* utilised an integrated gradients method to show which pixels corresponded to the most important clinical and radiomics features for prediction of overall survival in patients with HCC undergoing stereotactic body radiation therapy.^109^

## Outlook

### Overcoming obstacles on the way to clinical implementation

Even though AI carries much promise for changing future clinical practice, a number of issues must be addressed before broad implementation is possible. The problems of data standardisation, biases introduced through unrepresentative training data, and explainability of ML/DL algorithms have already been mentioned above. However, these issues are more concerned with model development, rather than deployment. Building up the necessary healthcare infrastructure and training medical personnel to sensibly use new technology are important cornerstones of the deployment side. To fully realise the benefits of Big Data, stakeholders must enforce and accelerate the digitisation of healthcare units. In that respect, whereas most radiology units in industrialised countries are fully digitised, most pathology departments are not. Nevertheless, we believe that digitised workflows will soon be adopted by pathology, permitting seamless integration and application of ML/DL tools amongst departments heavily dependent on imaging. At present, most AI tools are designed for a single specific task. In the future, we envision a standardised software suite that will incorporate many different plug-in options. Ideally, this software suite would be publicly available through an open-source project funded by government or independent healthcare institutions. This would avoid dependency on private companies and nudge industry to standardise its products, reducing the cost and the number of proprietary data formats and software solutions. Additionally, a single software platform would make it easier for medical staff to work with several applications and algorithms, hence reducing the investment in training.

### Multimodal input models for clinical decision making

**Fig. 3:**
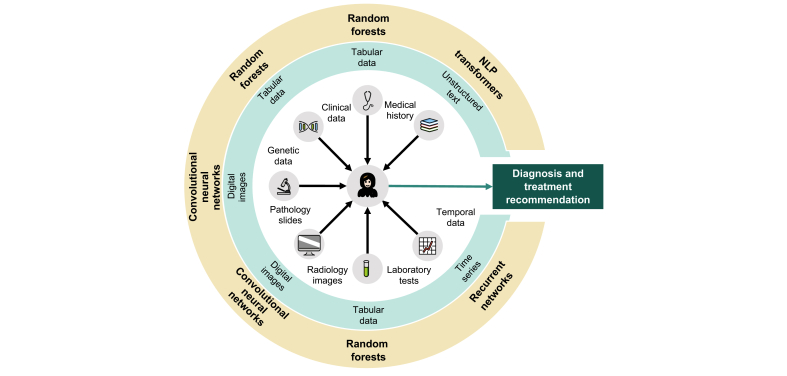
Data types in hepatology and multimodal learning. Inner area: data types routinely used for clinical decision making in hepatology. Blue circle: type of digital data. Yellow circle: suitable machine learning methods to analyse this data. NLP, natural language processing. Icon source: openmoji.org (CC-BY-SA 4.0 license).

Decision making in clinical routine is rarely based on a single data modality. Usually, healthcare providers integrate a number of different data types into clinical decisions. This is especially true in hepatology – a field in which it is rare for diseases to be directly observed and the differential diagnosis can be uncertain. For example, one of the most common hepatology consults is an incidental finding of elevated liver enzymes. Diagnosing the aetiology of this abnormality requires a battery of tests, including detailed clinical history, additional laboratory tests, ultrasound, and even histopathology. Supporting, and ultimately mimicking, human decision making in such complex tasks is currently out of reach for narrow and specialised AI systems. At present, different AI approaches are required to process various types of clinical input data (Fig. 3). Recently, there have been increasingly successful attempts to integrate multimodal data in non-medical fields,^110^ but such endeavours have not been systematically applied in a medical context beyond highly simplified laboratory conditions.^111^

### Interdisciplinary teaching and training

The medical profession will not be replaced by AI in the future, as the need to adapt to incomplete data, engagement in shared decision making with patients, and the ethical and legal obligation to assume responsibility will continue to remain in medicine. However, doctors can include the predictions of AI models in their recommendations and decisions, and thus use existing information more effectively. This incorporation of AI will require communication platforms, namely, user interfaces, dashboards, and innovative visualisation methods, to optimise the flow of information from AI to physicians. In order for AI to be widely adopted by the medical community, “digital literacy” needs to be a core medical competency. A necessary prerequisite for such “digital literacy” is basic knowledge of programming, which, in principle, can be learned by everyone. In the medical context, structured training programmes should be employed to teach programming. To that end, doctors must learn the necessary skills to use AI methods in research; validate algorithms in clinical studies; and critically question the benefits, data security, and possible biases of algorithms, even after regulatory approval. In our experience, it has been especially encouraging to witness medical students and young doctors who are earnestly interested in gaining a deeper understanding of AI and applying this technology to clinical problems. In time, this new generation of digital clinician scientists will acquire the rigorous training to advance AI research and pave the way for AI implementation into routine clinical workflows.

## Financial support

JNK is supported by the 10.13039/501100003107German Federal Ministry of Health (DEEP LIVER, ZMVI1-2520DAT111) and the Max-Eder-Programme of the German Cancer Aid (grant #70113864). TPS is supported by the 10.13039/501100003107German Federal Ministry of Health (DEEP LIVER, ZMVI1-2520DAT111). JC reports grants from the 10.13039/100000002National Institutes of Health (R01CA206180), Society of Interventional Oncology, 10.13039/100006098Radiological Society of North America, 10.13039/100004320Philips, Guerbet, 10.13039/100008497Boston Scientific and the 10.13039/100013043Yale Center for Clinical Investigation.

## Authors’ contributions

All authors wrote and critically revised this article and collectively made the decision to publish.

## Conflicts of interest

JNK declares consulting services for Owkin (France) and Panakeia (UK) and has received honoraria for scientific talks and participation in advisory boards by MSD, Eisai and Bayer. JC is a consultant for Guerbet, Bayer and Philips. VP is involved in a collaborative study with Owkin, France. DN and TPS declare no conflicts of interest.

Please refer to the accompanying ICMJE disclosure forms for further details.
