# Supplementary material for: Artificial intelligence in liver diseases: Improving diagnostics, prognostics and response prediction
Source: JHEP Rep. 2022 Feb 2;4(4):100443. doi: 10.1016/j.jhepr.2022.100443 (PMC8867112; doi:10.1016/j.jhepr.2022.100443)
Supplement: Multimedia component 1 [file mmc1.pdf]

Artificial intelligence in liver diseases: improving diagnostics, prognostics and response prediction

David Nam, Julius Chapiro, Valerie Paradis, Tobias Paul Seraphin, Jakob Nikolas Kather

Table of contents

Supplementary materials and methods..2

Table S1..3

Table S2..5

Supplementary references...13

Supplementary materials and methods

Literature search

On 8th September 2021 we performed a systematic literature search on the PubMed Database (https://pubmed.ncbi.nlm.nih.gov/) using the following search terms: “(("Artificial Intelligence") OR ("AI") OR ("Machine learning") OR ("Deep Learning")) AND (("Liver disease") OR ("NASH") OR ("NAFLD") OR ("HCC") OR ("Liver cancer")) AND ((Radiology) OR (Pathology)) AND ((diagnostics) OR (prognostics) OR (treatment))”. We excluded studies published before 1st January 2010. The search resulted in 686 articles which have been subsequently screened by abstract and title, using the systematic review tool Rayyan (https://www.rayyan.ai/) [112]. Our inclusion criteria were the following: 1. Human study, 2. Artificial intelligence/Deep Learning/Machine Learning methods used, 3. Related to pathology or radiology as clinical fields, 4. Method used for diagnostics, pronostics or treatment responses, 5. Dealing with liver diseases. The remaining 112 publications were then analyzed. No time restriction was applied. All search results were studies published between 7th January 2012 and 9th January 2021.

Data analysis for Figure 1

Figure 1A: “Others” includes countries with a maximum of two counts: Canada, Egypt, Hong Kong, Switzerland, Taiwan, Thailand, UK. Figure 1B: “Others” includes two technical papers and two studies combining diagnosis and prognosis. Figure 1C: “Other CLDs” includes diseases with a maximum of three counts. Figure 1D: “Others” includes input data with a maximum of one count.

Supplementary tables

Study	Year	Modality	HC/DL	Disease	Algorithm	Prediction	Highlight
Vanderbeck S et al. [23]	2014	H&E	HC	NAFLD	SVM	Diagnosis	Identify morphological tissue features
Vanderbeck S et al. [113]	2015	H&E	HC	NAFLD	SVM	Diagnosis	Detect lobular inflammation and hepatocellular ballooning
Li S et al.[32]	2017	N/A	DL	HCC	CNN	Diagnosis	HCC nuclei grading
Forlano R et al.[24]	2020	H&E, Sirius Red	HC	NAFLD	K-means	Diagnosis	Quantification of Steatosis, Inflammation, Ballooning and Fibrosis, diagnosing NASH
Gawrieh S et al. [28]	2020	Trichrome stain	HC	NAFLD	SVM	Diagnosis	Quantification and classification of hepatic fibrosis
Kiani A et al.[37]	2020	H&E	DL	HCC, CCC	CNN	Diagnosis	Classifying HCC vs. CCC and exploring the influence of the model on pathologists decisions
Leow WQ et al.[25]	2020	Unstained	HC	NASH	Linear regression, sequential feature selection	Diagnosis	Stratifying stage 1 and 2 NASH fibrosis
Liao H et al.[38]	2020	H&E	HC	HCC	RF, PCA	Diagnosis/Prognosis	Distinguish HCC from healthy liver and develop a risk score associated with overall survival
Roy M et al.[26]	2020	H&E	DL	Steatosis	CNN	Segmentation	Quantification of Steatosis
Saillard C et al.[40]	2020	H&E	DL	HCC	CNN	Prognosis	Survival after resection of HCC
Sun C et al.[35]	2020	H&E	DL	HCC	CNN	Diagnosis	Multiple instance learning model to diagnose HCC
Teramoto T et al.[20]	2020	H&E	HC	NAFLD	SVM	Diagnosis	Quantifying hepatocellular ballooning and classifying NASH and non-NASH NAFLD

Wang R et al.[34]	2020	Hyperspectral images	DL	HCC	CNN	Diagnosis	Diagnosing HCC from hyperspectral data of liver biopsies
Aatresh AA et al. [29]	2021	H&E	DL	HCC	CNN	Diagnosis	Classification of HCC in 4 histopathology classes
Khened M et al. [30]	2021	H&E	DL	HCC	CNN	Technical paper	End-to-end pipeline for Liver tissue segmentation and diagnosis of HCC
Lal S et al. [33]	2021	H&E	DL	Cancer	CNN	Segmentation	Segmentation of nuclei from liver cancer tissue
Pérez-Sanz F et al. [21]	2021	Sudan	HC	Steatosis	SVM, KNN, NN, Naive-Bayes	Diagnosis	Quantification of macrovesicular steatosis in donor livers
Qu H et al.[22]	2021	H&E, trichrome	DL	NAFLD	CNN	Diagnosis	Prediction of NAFLD Activity score and fibrosis stage
Roy M et al. [36]	2021	H&E	DL	HCC	CNN	Segmentation	HCC segmentation and classification of tumorous liver tissue
Saito A et al. [39]	2021	H&E	HC	HCC	SVM	Prognosis	Nuclei features for the prediction of recurrence after HCC resection
Shi JY et al.[41]	2021	H&E	DL	HCC	CNN	Prognosis	Development of a tumor risk score evaluating patients' outcomes
Taylor-Weiner A et al. [27]	2021	H&E	DL	NASH	CNN	Prognosis	Quantification of morphological features of NASH, fibrosis staging and prediction of disease progression
Wang X et al. [31]	2021	H&E	DL	HCC	CNN	Segmentation	Segmentation of HCC in liver tissue
Yamashita R et al. [42]	2021	H&E	DL	HCC	CNN	Prognosis	Prediction of postsurgical recurrence in HCC

Table S1. AI studies in liver histopathology.

Study	Year	Modality	HC/DL	Disease	Algorithm	Prediction	Application
Acharya UR et al. [114]	2012	Ultrasound	HC	FLD	Decision tree classifier	Diagnosis	Classification of normal vs. abnormal livers affected by fatty liver disease
Jiang H et al. [115]	2013	CT	HC	Cancer	SVM, particle swarm and local optimization	Diagnosis	Classification of normal liver, liver cancer, liver cirrhosis, and liver cyst
Kadoury S et al. [116]	2015	CT	HC	Cancer	Discriminant Grassmanian manifolds, conditional random fields	Segmentation	Metastatic liver tumor segmentation
Huang L et al. [117]	2016	CT	HC	Cancer, hemangioma	Single-block linear detection	Segmentation	Liver segmentation
Conze PH et al. [118]	2017	CT	HC	HCC	RF, hierarchical multi-scale tree	Segmentation	HCC tumor segmentation
Gatos I et al. [119]	2017	Ultrasound	HC	CLD	Stepwise regression analysis, SVM	Diagnosis	Classification of healthy vs. chronic liver disease-affected livers
Vorontsov E et al. [120]	2017	CT	DL	Tumor	Multilayer perceptron NN, deformable model	Segmentation	Metastatic liver tumor segmentation
Abajian A et al. [85]	2018	MRI + clinical data	HC	HCC	LR, RF	Prognosis	Prediction of response to TACE in HCC patients
Acharya UR et al. [121]	2018	Ultrasound	HC	Lesions	Bidirectional empirical mode decomposition, particle swarm optimization, PNN	Diagnosis	Classification of normal liver as well as benign and malignant focal liver lesions
Biswas M et al. [68]	2018	Ultrasound	DL	FLD	CNN	Diagnosis	Detection of and risk stratification for fatty liver disease
Byra M et al. [69]	2018	Ultrasound	DL	FLD	CNN, SVM, LASSO linear regression	Diagnosis	Detection of fatty liver disease and quantification of hepatic steatosis
Choi KJ et al. [73]	2018	CT	DL	Fibrosis	CNN	Segmentation/Diagnosis	Fibrosis stage classification
Li X et al. [64]	2018	CT	DL	Lesions	CNN	Segmentation	Liver and liver lesion segmentation

Ahn SH et al. [122]	2019	CT	DL	Organ segmentation	CNN	Segmentation	Segmentation of liver and organs at risk during radiation planning
Feng ST et al. [81]	2019	MRI	HC	HCC	LASSO LR, LR	Diagnosis	Preoperative MVI prediction in HCC patients who underwent curative hepatectomy
Gatos I et al. [123]	2019	Ultrasound	DL	Fibrosis	CNN	Diagnosis	Mask separating high and low temporal stability areas, classification of fibrosis in chronic liver disease
Graffy PM et al. [70]	2019	CT	DL	Steatosis	CNN	Segmentation	Quantification of liver steatosis
Guo D et al. [124]	2019	CT + clinical data	HC	HCC	LASSO, Cox	Prognosis	Prediction of HCC recurrence after liver transplantation
Hamm CA et al. [76]	2019	MRI	DL	Lesions	CNN	Diagnosis	Classification of hepatic lesions
He L et al. [125]	2019	MRI + clinical data	HC	Fibrosis	LASSO, SVM	Diagnosis	Classification of liver stiffness
Huo Y et al. [126]	2019	CT	DL	NAFLD	CNN	Segmentation/Diagnosis	Quantification of liver attenuation and detection of NAFLD
Jansen MJA et al. [127]	2019	MRI + clinical data	HC	Lesions	Extremely randomized trees classifier	Diagnosis	Classification of liver lesions
Ji GW et al. [128]	2019	CT + clinical data + radiological data	HC	HCC	MRMR, RSF, LASSO Cox, unsupervised hierarchical clustering, Cox	Prognosis	Prediction of HCC recurrence after curative resection
Nayak A et al. [129]	2019	CT	HC	HCC	LR, SVM	Segmentation/Diagnosis	Liver segmentation, classification of healthy, cirrhosis, and cirrhosis with HCC livers
Ouhmich F et al. [130]	2019	CT	DL	HCC	CNN	Segmentation	Segmentation of liver parenchyma, active and necrotic tumor
Oyama A et al. [131]	2019	MRI	HC	HCC, metastasis, hemangioma	Texture analysis and LDA, topological data analysis and XGBoost	Diagnosis	Classification of HCC, metastasis, and hepatic hemangioma

Shan QY et al. [132]	2019	CT	HC	HCC	LASSO linear regression	Prognosis	Prediction of early HCC recurrence after curative resection or ablation
Wang CJ et al. [107]	2019	MRI	DL	Lesions	CNN	Diagnosis	Follow-up study to Hamm CA et al. [76] for identification of features contributing to lesion classification, generation of feature maps, and calculation of feature relevance scores
Wang K et al. [133]	2019	CT + MRI	DL	None	CNN	Segmentation	Liver segmentation, computation of liver volume and hepatic proton density fat fraction
Wang K et al. [72]	2019	Ultrasound	DL	Fibrosis	CNN	Diagnosis	Fibrosis stage classification
Wang W et al. [134]	2019	CT + clinical data	DL	HCC	CNN	Prognosis	Prediction of early HCC recurrence after resection
Yang DW et al. [135]	2019	MRI	DL	HCC	CNN	Diagnosis	Diagnosis of HCC pathologic grade
Ahmed Y et al. [136]	2020	MRI	HC	Fibrosis	SVM	Diagnosis	Classification of normal or fibrotic liver
Budai BK et al. [137]	2020	CT	HC	Fibrosis	K-means, hierarchical cluster analysis, linear regression, PCA,, RF, SVM, RFE	Diagnosis	Classification of low-grade and high-grade fibrosis
Cao SE et al. [138]	2020	CT	DL	Focal liver lesions	CNN	Diagnosis	Classification of focal liver lesions
Chen WF et al. [139]	2020	CT	DL	Tumor	CNN	Segmentation	Liver and lesion segmentation
Doman K et al. [140]	2020	CT	DL	Metastasis	Lesion image generation with Poisson blending, CT value distribution, and DCGANs; CNN	Technical Paper/Diagnosis	Detection of liver metastasis
Han A et al.[71]	2020	Ultrasound	DL	NAFLD	CNN	Diagnosis	NAFLD diagnosis and hepatic fat quantification
Hu HT et al. [89]	2020	CT	HC	HCC	LASSO	Prognosis	Prediction of early HCC recurrence after resection or ablation

Kagadis GC et al. [141]	2020	Ultrasound	DL	CLD	CNN	Diagnosis	Fibrosis stage classification
Kim J et al. [142]	2020	MRI	DL	HCC	CNN	Diagnosis	HCC detection
Li Q et al. [143]	2020	CT	DL	Fibrosis	CNN	Diagnosis	Fibrosis stage classification
Liang W et al. [144]	2020	CT + MRI + clinical data	HC	HCC, hepatic epithelioid angiomyolipoma, FNH	Mutual information, RF, LR	Diagnosis	Classification of hepatic epithelioid angiomyolipoma from HCC and FNH
Liu QP et al. [145]	2020	CT + clinical data + radiological data	HC, DL	HCC	RF, SVM, DAE, time-varying DL algorithm, Cox	Diagnosis/Prognosis	Predictions for MVI, Edmondson-Steiner grade, and prognosis/survival after TACE
Mao B et al. [146]	2020	CT + clinical data	HC	HCC	RFE, XGBoost	Diagnosis	HCC pathologic grade prediction
Mokrane FZ et al. [147]	2020	CT	HC	HCC	KNN, RF	Diagnosis	HCC diagnosis in a cirrhotic background with indeterminate liver nodules on, HCCrisk stratification
Nebbia G et al. [148]	2020	MRI	HC	HCC	LASSO, SVM	Diagnosis	MVI prediction
Oezdemir I et al. [149]	2020	Ultrasound	HC	HCC	Distance-weighted discrimination method	Prognosis	Prediction of response to TACE
Peng J et al. [87]	2020	CT	DL	HCC	CNN	Prognosis	Prediction of response to TACE
Ponnoprat D et al. [150]	2020	CT	DL, HC	HCC, CCC	CNN, SVM	Segmentation/Diagnosis	Classification of HCC and intrahepatic cholangiocarcinoma
Schawkat K et al. [151]	2020	MRI	HC	Fibrosis	PCA, SVM	Diagnosis	Fibrosis stage classification
Shi W et al. [152]	2020	CT	DL	HCC	CNN	Diagnosis	Classification of HCC from other focal liver lesions
Son JH et al. [153]	2020	CT	DL	CLD	CNN	Segmentation/Diagnosis	Fibrosis stage classification, correlation of liver and spleen volumetrics with fibrosis stage

Wei L et al. [154]	2020	CT + PET + dosimetry data	HC	Lesions	LASSO LR, LASSO Cox	Prognosis	Prediction of lesion-level response to radioembolization and lesion-level progression
Wu Y et al. [155]	2020	MRI	DL	HCC	CNN	Diagnosis	Classification of LI-RADS grade 3 from LI-RADS grade 4 and 5 lesions
Yang Q et al. [156]	2020	Ultrasound + clinical data + radiological data	DL	Lesions	CNN, LR	Diagnosis	Classification of benign and malignant focal liver lesions
Zhang L et al. [157]	2020	CT + clinical data	DL	HCC	CNN, MRMR, Elastic Net, Cox	Prognosis	Overall survival prediction in HCC patients treated with TACE and sorafenib
Zhen SH et al. [108]	2020	MRI + clinical data	DL	Tumor	CNN	Diagnosis	Classification of liver tumors
Bousabarah K et al. [65]	2021	MRI	DL + HC	HCC	CNN, RF	Segmentation	Segmentation of liver and HCC
Byra M et al. [158]	2021	Ultrasound	DL	NAFLD	CNN, LR, LASSO	Diagnosis	Diagnosis of fatty liver and advanced steatosis, quantification of proton density fat fraction
Che H et al. [159]	2021	Ultrasound	DL	NAFLD	CNN	Diagnosis	NAFLD classification
Chen W et al. [160]	2021	CT	HC	HCC	LASSO, SVM	Diagnosis	Differentiation between low-grade and high-grade HCC
Chen Y et al. [161]	2021	MRI	DL	HCC	CNN, GDBT, Cox	Segmentation/Diagnosis/Prognosis	Preoperative prediction of cytokeratin 19 expression and analysis of recurrence-free survival
Chen Y et al. [162]	2021	MRI	HC	HCC	LASSO, KNN, SVM, LR, XGBoost	Diagnosis	MVI prediction
Das A et al. [163]	2021	Ultrasound	HC	NAFLD	SVM, multi-layered perceptron NN, XGBoost, ensemble learning	Diagnosis	Classification of NAFLD from normal liver
Gao F et al. [164]	2021	MRI	DL + HC	HCC	CNN, LASSO LR, ensemble learning	Diagnosis	MVI prediction

He K et al. [66]	2021	CT	DL	Tumor	CNN	Segmentation	Segmentation of tumor and ablation zone
Jiang YQ et al. [82]	2021	CT + clinical data+ radiological data	DL + HC	HCC	CNN, XGBoost	Diagnosis	MVI prediction
Jin Z et al. [88]	2021	CT + clinical data + radiological data	HC	HCC	LDA, Bayes, LR	Prognosis	Prediction of extrahepatic spread and macrovascular invasion in HCC patients who underwent TACE
Kim DW et al. [165]	2021	CT	DL	Lesions	CNN	Segmentation/Diagnosis	Detection of primary liver tumors
Li H et al. [166]	2021	MRI + clinical data	DL	CLD	CNN	Diagnosis	Liver stiffness classification
Liu P et al. [167]	2021	CT	HC	HCC	LASSO, LR	Diagnosis	MVI prediction
Liu X et al. [168]	2021	CT + MRI	HC	HCC, CCC	PCA, SVM	Diagnosis	Differentiation of combined HCC and CCC, CCC, as well as CCC
Nakai H et al. [169]	2021	CT	DL	HCC, CCC	CNN	Diagnosis	Differentiation of moderately differentiated HCC, poorly differentiated HCC, and intrahepatic cholangiocarcinoma
Nowak S et al. [170]	2021	MRI	DL	Cirrhosis	CNN	Segmentation/Diagnosis	Diagnosis of cirrhosis
Oestmann PM et al. [77]	2021	MRI	DL	HCC	CNN	Diagnosis	Lesion characterization, classification of typical HCC and atypical HCC from non-HCC
Pickhardt PJ et al. [171]	2021	CT	DL	NAFLD	CNN	Segmentation	Steatosis characterization
Sheng R et al. [172]	2021	MRI	DL	HCC	CNN	Segmentation/Diagnosis	Semi-automation of LI-RADS grading
Song D et al. [83]	2021	MRI + clinical data	DL	HCC	CNN	Diagnosis	MVI status and MVI grade prediction

Tiyarattanachai T et al. [173]	2021	Ultrasound	DL	Lesions	CNN	Diagnosis	Diagnosis of liver lesions
Wan Y et al. [174]	2021	MRI	DL	Lesions	CNN	Diagnosis	Diagnosis of liver lesions
Wang M et al. [175]	2021	CT	DL	HCC	CNN	Diagnosis	Diagnosis of HCC
Wei L et al. [109]	2021	CT + clinical data	DL + HC	HCC	CNN, VAE	Prognosis	Survival in HCC patients treated with SBRT
Zhang Y et al. [176]	2021	MRI	DL	HCC	CNN	Diagnosis	MVI prediction
Zheng R et al. [177]	2021	MRI	DL + HC	Fibrosis	CNN, LASSO LR, Gaussian Naive Bayes, SVM, LR	Segmentation/Diagnosis	Fibrosis stage classification
Zhou B et al. [67]	2021	CT + MRI	DL	HCC	CNN	Segmentation	Multimodal registration and segmentation for TACE
Zhou W et al. [84]	2021	MRI	DL	HCC	CNN	Diagnosis	MVI prediction
He T et al. [14]	2021	MRI + WSI + clinical data	DL	HCC	CNN, NLP, RBF NN, multilayer perceptron NN	Segmentation/Prognosis	Recurrence of HCC after liver transplantation

Table S2. AI studies in liver radiology.

Abbreviations for Supplementary Tables S1 and S2:

CCC = cholangiocellular carcinoma

CLD = chronic liver disease

CNN = convolutional neural network

CT = computed tomography

DAE = deep auto-encoder

DCGAN = deep convolutional generative adversarial networks

DL = deep learning

FLD = fatty liver disease

FNH = focal nodular hyperplasia

GDBT = gradient boosting tree

H&E = hematoxylin and eosin

HC = handcrafted features

HCC = hepatocellular carcinoma

KNN = K-nearest neighbors

LASSO = least absolute shrinkage and selection operator

LDA = linear discriminant analysis

LR = logistic regression

MRI = magnetic resonance imaging

MRMR = maximum relevance minimum redundancy

MVI = microvascular invasion

NAFLD = nonalcoholic fatty liver disease

NASH = nonalcoholic steatohepatitis

NLP = natural language processing

NN = neural network

PCA = principal component analysis

PNN = probabilistic neural network

RBF NN = radial basis function neural network

RF = random forest

RFE = recursive feature elimination

SVM = support vector machine

TACE = transarterial chemoembolization

VAE = variational auto-encoder

XGBoost= eXtreme Gradient Boosting

Additional references for supplementary tables

[112] Ouzzani M, Hammady H, Fedorowicz Z, Elmagarmid A. Rayyan-a web and mobile app for systematic reviews. Syst Rev 2016;5:210.
[113] Vanderbeck S, Bockhorst J, Kleiner D, Komorowski R, Chalasani N, Gawrieh S. Automatic quantification of lobular inflammation and hepatocyte ballooning in nonalcoholic fatty liver disease liver biopsies. Hum Pathol 2015;46:767–75.
[114] Acharya UR, Sree SV, Ribeiro R, Krishnamurthi G, Marinho RT, Sanches J, et al. Data mining framework for fatty liver disease classification in ultrasound: a hybrid feature extraction paradigm. Med Phys 2012;39:4255–64.
[115] Jiang H, Zheng R, Yi D, Zhao D. A novel multiinstance learning approach for liver cancer recognition on abdominal CT images based on CPSO-SVM and IO. Comput Math Methods Med 2013;2013:434969.
[116] Kadoury S, Vorontsov E, Tang A. Metastatic liver tumour segmentation from discriminant Grassmannian manifolds. Phys Med Biol 2015;60:6459–78.
[117] Huang L, Weng M, Shuai H, Huang Y, Sun J, Gao F. Automatic Liver Segmentation from CT Images Using Single-Block Linear Detection. Biomed Res Int 2016;2016:9420148.
[118] Conze P-H, Noblet V, Rousseau F, Heitz F, de Blasi V, Memeo R, et al. Scale-adaptive supervoxel-based random forests for liver tumor segmentation in dynamic contrast-enhanced CT scans. Int J Comput Assist Radiol Surg 2017;12:223–33.
[119] Gatos I, Tsantis S, Spiliopoulos S, Karnabatidis D, Theotokas I, Zoumpoulis P, et al. A Machine-Learning Algorithm Toward Color Analysis for Chronic Liver Disease Classification, Employing Ultrasound Shear Wave Elastography. Ultrasound Med Biol 2017;43:1797–810.
[120] Vorontsov E, Tang A, Roy D, Pal CJ, Kadoury S. Metastatic liver tumour segmentation with a neural network-guided 3D deformable model. Med Biol Eng Comput 2017;55:127–39.
[121] Acharya UR, Koh JEW, Hagiwara Y, Tan JH, Gertych A, Vijayananthan A, et al. Automated diagnosis of focal liver lesions using bidirectional empirical mode decomposition features. Comput Biol Med 2018;94:11–8.
[122] Ahn SH, Yeo AU, Kim KH, Kim C, Goh Y, Cho S, et al. Comparative clinical evaluation of atlas and deep-learning-based auto-segmentation of organ structures in liver cancer. Radiat Oncol 2019;14:213.
[123] Gatos I, Tsantis S, Spiliopoulos S, Karnabatidis D, Theotokas I, Zoumpoulis P, et al. Temporal stability assessment in shear wave elasticity images validated by deep learning neural network for chronic liver disease fibrosis stage assessment. Med Phys 2019;46:2298–309.
[124] Guo D, Gu D, Wang H, Wei J, Wang Z, Hao X, et al. Radiomics analysis enables recurrence prediction for hepatocellular carcinoma after liver transplantation. Eur J Radiol 2019;117:33–40.
[125] He L, Li H, Dudley JA, Maloney TC, Brady SL, Somasundaram E, et al. Machine Learning Prediction of Liver Stiffness Using Clinical and T2-Weighted MRI Radiomic Data. AJR Am J Roentgenol 2019;213:592–601.
[126] Huo Y, Terry JG, Wang J, Nair S, Lasko TA, Freedman BI, et al. Fully automatic liver attenuation estimation combing CNN segmentation and morphological operations. Med Phys 2019;46:3508–19.
[127] Jansen MJA, Kuijf HJ, Veldhuis WB, Wessels FJ, Viergever MA, Pluim JPW.

Automatic classification of focal liver lesions based on MRI and risk factors. PLoS One 2019;14:e0217053.
[128] Ji G-W, Zhu F-P, Xu Q, Wang K, Wu M-Y, Tang W-W, et al. Machine-learning analysis of contrast-enhanced CT radiomics predicts recurrence of hepatocellular carcinoma after resection: A multi-institutional study. EBioMedicine 2019;50:156–65.
[129] Nayak A, Baidya Kayal E, Arya M, Culli J, Krishan S, Agarwal S, et al. Computer-aided diagnosis of cirrhosis and hepatocellular carcinoma using multi-phase abdomen CT. Int J Comput Assist Radiol Surg 2019;14:1341–52.
[130] Ouhmich F, Agnus V, Noblet V, Heitz F, Pessaux P. Liver tissue segmentation in multiphase CT scans using cascaded convolutional neural networks. Int J Comput Assist Radiol Surg 2019;14:1275–84.
[131] Oyama A, Hiraoka Y, Obayashi I, Saikawa Y, Furui S, Shiraishi K, et al. Hepatic tumor classification using texture and topology analysis of non-contrast-enhanced three-dimensional T1-weighted MR images with a radiomics approach. Sci Rep 2019;9:8764.
[132] Shan Q-Y, Hu H-T, Feng S-T, Peng Z-P, Chen S-L, Zhou Q, et al. CT-based peritumoral radiomics signatures to predict early recurrence in hepatocellular carcinoma after curative tumor resection or ablation. Cancer Imaging 2019;19:11.
[133] Wang K, Mamidipalli A, Retson T, Bahrami N, Hasenstab K, Blansit K, et al. Automated CT and MRI Liver Segmentation and Biometry Using a Generalized Convolutional Neural Network. Radiol Artif Intell 2019;1. https://doi.org/10.1148/ryai.2019180022.
[134] Wang W, Chen Q, Iwamoto Y, Han X, Zhang Q, Hu H, et al. Deep Learning-Based Radiomics Models for Early Recurrence Prediction of Hepatocellular Carcinoma with Multi-phase CT Images and Clinical Data. Conf Proc IEEE Eng Med Biol Soc 2019;2019:4881–4.
[135] Yang D-W, Jia X-B, Xiao Y-J, Wang X-P, Wang Z-C, Yang Z-H. Noninvasive Evaluation of the Pathologic Grade of Hepatocellular Carcinoma Using MCF-3DCNN: A Pilot Study. Biomed Res Int 2019;2019:9783106.
[136] Ahmed Y, Hussein RS, Basha TA, Khalifa AM, Ibrahim AS, Abdelmoaty AS, et al. Detecting liver fibrosis using a machine learning-based approach to the quantification of the heart-induced deformation in tagged MR images. NMR Biomed 2020;33:e4215.
[137] Budai BK, Tóth A, Borsos P, Frank VG, Shariati S, Fejér B, et al. Three-dimensional CT texture analysis of anatomic liver segments can differentiate between low-grade and high-grade fibrosis. BMC Med Imaging 2020;20:108.
[138] Cao S-E, Zhang L-Q, Kuang S-C, Shi W-Q, Hu B, Xie S-D, et al. Multiphase convolutional dense network for the classification of focal liver lesions on dynamic contrast-enhanced computed tomography. World J Gastroenterol 2020;26:3660–72.
[139] Chen W-F, Ou H-Y, Liu K-H, Li Z-Y, Liao C-C, Wang S-Y, et al. In-Series U-Net Network to 3D Tumor Image Reconstruction for Liver Hepatocellular Carcinoma Recognition. Diagnostics (Basel) 2020;11. https://doi.org/10.3390/diagnostics11010011.
[140] Doman K, Konishi T, Mekada Y. Lesion Image Synthesis Using DCGANs for Metastatic Liver Cancer Detection. Adv Exp Med Biol 2020;1213:95–106.
[141] Kagadis GC, Drazinos P, Gatos I, Tsantis S, Papadimitroulas P, Spiliopoulos S, et al. Deep learning networks on chronic liver disease assessment with fine-tuning of shear wave elastography image sequences. Phys Med Biol 2020;65:215027.
[142] Kim J, Min JH, Kim SK, Shin S-Y, Lee MW. Detection of Hepatocellular Carcinoma in Contrast-Enhanced Magnetic Resonance Imaging Using Deep Learning Classifier: A Multi-Center Retrospective Study. Sci Rep 2020;10:9458.

[143] Li Q, Yu B, Tian X, Cui X, Zhang R, Guo Q. Deep residual nets model for staging liver fibrosis on plain CT images. Int J Comput Assist Radiol Surg 2020;15:1399–406.
[144] Liang W, Shao J, Liu W, Ruan S, Tian W, Zhang X, et al. Differentiating Hepatic Epithelioid Angiomyolipoma From Hepatocellular Carcinoma and Focal Nodular Hyperplasia via Radiomics Models. Front Oncol 2020;10:564307.
[145] Liu Q-P, Xu X, Zhu F-P, Zhang Y-D, Liu X-S. Prediction of prognostic risk factors in hepatocellular carcinoma with transarterial chemoembolization using multi-modal multi-task deep learning. EClinicalMedicine 2020;23:100379.
[146] Mao B, Zhang L, Ning P, Ding F, Wu F, Lu G, et al. Preoperative prediction for pathological grade of hepatocellular carcinoma via machine learning-based radiomics. Eur Radiol 2020;30:6924–32.
[147] Mokrane F-Z, Lu L, Vavasseur A, Otal P, Peron J-M, Luk L, et al. Radiomics machine-learning signature for diagnosis of hepatocellular carcinoma in cirrhotic patients with indeterminate liver nodules. Eur Radiol 2020;30:558–70.
[148] Nebbia G, Zhang Q, Arefan D, Zhao X, Wu S. Pre-operative Microvascular Invasion Prediction Using Multi-parametric Liver MRI Radiomics. J Digit Imaging 2020;33:1376–86.
[149] Oezdemir I, Wessner CE, Shaw C, Eisenbrey JR, Hoyt K. Tumor Vascular Networks Depicted in Contrast-Enhanced Ultrasound Images as a Predictor for Transarterial Chemoembolization Treatment Response. Ultrasound Med Biol 2020;46:2276–86.
[150] Ponnoprat D, Inkeaw P, Chaijaruwanich J, Traisathit P, Sripan P, Inmutto N, et al. Classification of hepatocellular carcinoma and intrahepatic cholangiocarcinoma based on multi-phase CT scans. Med Biol Eng Comput 2020;58:2497–515.
[151] Schawkat K, Ciritsis A, von Ulmenstein S, Honcharova-Biletska H, Jüngst C, Weber A, et al. Diagnostic accuracy of texture analysis and machine learning for quantification of liver fibrosis in MRI: correlation with MR elastography and histopathology. Eur Radiol 2020;30:4675–85.
[152] Shi W, Kuang S, Cao S, Hu B, Xie S, Chen S, et al. Deep learning assisted differentiation of hepatocellular carcinoma from focal liver lesions: choice of four-phase and three-phase CT imaging protocol. Abdom Radiol (NY) 2020;45:2688–97.
[153] Son JH, Lee SS, Lee Y, Kang B-K, Sung YS, Jo S, et al. Assessment of liver fibrosis severity using computed tomography-based liver and spleen volumetric indices in patients with chronic liver disease. Eur Radiol 2020;30:3486–96.
[154] Wei L, Cui C, Xu J, Kaza R, El Naqa I, Dewaraja YK. Tumor response prediction in 90Y radioembolization with PET-based radiomics features and absorbed dose metrics. EJNMMI Phys 2020;7:74.
[155] Wu Y, White GM, Cornelius T, Gowdar I, Ansari MH, Supanich MP, et al. Deep learning LI-RADS grading system based on contrast enhanced multiphase MRI for differentiation between LR-3 and LR-4/LR-5 liver tumors. Ann Transl Med 2020;8:701.
[156] Yang Q, Wei J, Hao X, Kong D, Yu X, Jiang T, et al. Improving B-mode ultrasound diagnostic performance for focal liver lesions using deep learning: A multicentre study. EBioMedicine 2020;56:102777.
[157] Zhang L, Xia W, Yan Z-P, Sun J-H, Zhong B-Y, Hou Z-H, et al. Deep Learning Predicts Overall Survival of Patients With Unresectable Hepatocellular Carcinoma Treated by Transarterial Chemoembolization Plus Sorafenib. Front Oncol 2020;10:593292.
[158] Byra M, Han A, Boehringer AS, Zhang YN, O'Brien WD Jr, Erdman JW Jr, et al. Liver Fat Assessment in Multiview Sonography Using Transfer Learning With Convolutional Neural Networks. J Ultrasound Med 2021. https://doi.org/10.1002/jum.15693.
[159] Che H, Brown LG, Foran DJ, Nosher JL, Hacihaliloglu I. Liver disease classification

from ultrasound using multi-scale CNN. Int J Comput Assist Radiol Surg 2021;16:1537–48.
[160] Chen W, Zhang T, Xu L, Zhao L, Liu H, Gu LR, et al. Radiomics Analysis of Contrast-Enhanced CT for Hepatocellular Carcinoma Grading. Front Oncol 2021;11:660509.
[161] Chen Y, Chen J, Zhang Y, Lin Z, Wang M, Huang L, et al. Preoperative Prediction of Cytokeratin 19 Expression for Hepatocellular Carcinoma with Deep Learning Radiomics Based on Gadoxetic Acid-Enhanced Magnetic Resonance Imaging. J Hepatocell Carcinoma 2021;8:795–808.
[162] Chen Y, Xia Y, Tolat PP, Long L, Jiang Z, Huang Z, et al. Comparison of Conventional Gadoxetate Disodium-Enhanced MRI Features and Radiomics Signatures With Machine Learning for Diagnosing Microvascular Invasion. AJR Am J Roentgenol 2021;216:1510–20.
[163] Das A, Connell M, Khetarpal S. Digital image analysis of ultrasound images using machine learning to diagnose pediatric nonalcoholic fatty liver disease. Clin Imaging 2021;77:62–8.
[164] Gao F, Qiao K, Yan B, Wu M, Wang L, Chen J, et al. Hybrid network with difference degree and attention mechanism combined with radiomics (H-DARnet) for MVI prediction in HCC. Magn Reson Imaging 2021;83:27–40.
[165] Kim DW, Lee G, Kim SY, Ahn G, Lee J-G, Lee SS, et al. Deep learning-based algorithm to detect primary hepatic malignancy in multiphase CT of patients at high risk for HCC. Eur Radiol 2021;31:7047–57.
[166] Li H, He L, Dudley JA, Maloney TC, Somasundaram E, Brady SL, et al. DeepLiverNet: a deep transfer learning model for classifying liver stiffness using clinical and T2-weighted magnetic resonance imaging data in children and young adults. Pediatr Radiol 2021;51:392–402.
[167] Liu P, Tan X-Z, Zhang T, Gu Q-B, Mao X-H, Li Y-C, et al. Prediction of microvascular invasion in solitary hepatocellular carcinoma ≤ 5 cm based on computed tomography radiomics. World J Gastroenterol 2021;27:2015–24.
[168] Liu X, Khalvati F, Namdar K, Fischer S, Lewis S, Taouli B, et al. Can machine learning radiomics provide pre-operative differentiation of combined hepatocellular cholangiocarcinoma from hepatocellular carcinoma and cholangiocarcinoma to inform optimal treatment planning? Eur Radiol 2021;31:244–55.
[169] Nakai H, Fujimoto K, Yamashita R, Sato T, Someya Y, Taura K, et al. Convolutional neural network for classifying primary liver cancer based on triple-phase CT and tumor marker information: a pilot study. Jpn J Radiol 2021;39:690–702.
[170] Nowak S, Mesropyan N, Faron A, Block W, Reuter M, Attenberger UI, et al. Detection of liver cirrhosis in standard T2-weighted MRI using deep transfer learning. Eur Radiol 2021;31:8807–15.
[171] Pickhardt PJ, Blake GM, Graffy PM, Sandfort V, Elton DC, Perez AA, et al. Liver Steatosis Categorization on Contrast-Enhanced CT Using a Fully Automated Deep Learning Volumetric Segmentation Tool: Evaluation in 1204 Healthy Adults Using Unenhanced CT as a Reference Standard. AJR Am J Roentgenol 2021;217:359–67.
[172] Sheng R, Huang J, Zhang W, Jin K, Yang L, Chong H, et al. A Semi-Automatic Step-by-Step Expert-Guided LI-RADS Grading System Based on Gadoxetic Acid-Enhanced MRI. J Hepatocell Carcinoma 2021;8:671–83.
[173] Tiyarattanachai T, Apiparakoon T, Marukatat S, Sukcharoen S, Geratikornsupuk N, Anukulkarnkusol N, et al. Development and validation of artificial intelligence to detect and diagnose liver lesions from ultrasound images. PLoS One 2021;16:e0252882.
[174] Wan Y, Zheng Z, Liu R, Zhu Z, Zhou H, Zhang X, et al. A Multi-Scale and Multi-Level

Fusion Approach for Deep Learning-Based Liver Lesion Diagnosis in Magnetic Resonance Images with Visual Explanation. Life 2021;11. https://doi.org/10.3390/life11060582.
[175] Wang M, Fu F, Zheng B, Bai Y, Wu Q, Wu J, et al. Development of an AI system for accurately diagnose hepatocellular carcinoma from computed tomography imaging data. Br J Cancer 2021;125:1111–21.
[176] Zhang Y, Lv X, Qiu J, Zhang B, Zhang L, Fang J, et al. Deep Learning With 3D Convolutional Neural Network for Noninvasive Prediction of Microvascular Invasion in Hepatocellular Carcinoma. J Magn Reson Imaging 2021;54:134–43.
[177] Zheng R, Shi C, Wang C, Shi N, Qiu T, Chen W, et al. Imaging-Based Staging of Hepatic Fibrosis in Patients with Hepatitis B: A Dynamic Radiomics Model Based on Gd-EOB-DTPA-Enhanced MRI. Biomolecules 2021;11. https://doi.org/10.3390/biom11020307.
